# Optimization and Comparative Study of Non-Pressurized Shell-and-Tube Latent Heat Storage for Air-Source Heat Pump Systems: Numerical and Experimental Investigation

**DOI:** 10.3390/ma19102014

**Published:** 2026-05-12

**Authors:** Weilin Li, Yuguo Fu, Hanrui Wang, Xingtao Zhang

**Affiliations:** School of Civil Engineering, Zhengzhou University, Zhengzhou 450001, China

**Keywords:** latent heat storage, shell-and-tube heat exchanger, air-source heat pump system, finned tube enhanced heat transfer, corrugated tube enhanced heat transfer

## Abstract

**Highlights:**

- A novel non-pressurized shell-and-tube latent heat storage (NP-LHS) device coupled with an air-source heat pump is proposed and validated.
- Finned tubes drastically outperform corrugated tubes for low-conductivity PCMs, achieving a 92.5% filling rate and reducing the complete melting time to 180 min.
- Full-cycle experiments demonstrate the finned tube NP-LHS successfully achieves a 98.0% peak-shaving power reduction rate while maintaining indoor thermal comfort.

**Abstract:**

To mitigate the spatiotemporal mismatch between renewable energy supply and building heating demand, this study proposes a novel non-pressurized shell-and-tube latent heat storage (NP-LHS) device coupled with an air-source heat pump (ASHP) system. To overcome the inherent low thermal conductivity of organic phase change materials (PCMs), the thermal performances of plain, corrugated, and finned tubes were systematically compared using both computational fluid dynamics (CFD) simulations and full-scale experiments. Numerical results indicate that the optimal tube spacing ratio ranges from 1.0 to 1.5. Among the evaluated geometries, the finned tube configuration exhibited superior comprehensive performance. It achieved an exceptionally high PCM volume fraction of 92.5% and dramatically reduced the complete melting time to 180 min—significantly faster than both corrugated (280 min) and bare tubes—while attaining a higher terminal temperature. Full-cycle dynamic experiments further demonstrated that integrating the finned tube NP-LHS into the ASHP system yielded a peak-shaving power reduction rate of 98.0%, effectively maintaining indoor thermal comfort. These findings conclude that expanding the conductive surface area via fins is practically more effective than inducing fluid turbulence for low-conductivity PCMs in non-pressurized storage applications.

## 1. Introduction

Recent statistics indicate that building operations contribute approximately 34% of global energy-related carbon dioxide emissions [1]. As an important component of modern comfortable living, The cooling and heating systems of the building can account for up to 50% of energy consumption in buildings [2,3]. Renewable energy can serve as a key low-carbon solution [4], but there is a spatiotemporal mismatch between the intermittency and volatility of its power generation and the continuous energy demand of buildings [5,6]. Thermal energy storage (TES) technology is regarded as one of the key approaches to resolving this contradiction and enhancing energy utilization efficiency [7]. The air-source heat pump system (ASHP) utilizes air as the heat source or cooling source. It has numerous advantages, such as a high energy efficiency ratio, the ability to utilize renewable energy sources, and the potential to reduce operating costs [8]. Developing a coupled system of building TES devices and ASHP can enhance the utilization of renewable energy. This coupled system can efficiently store heat through the heat pump when renewable energy is abundant and release heat during peak demand, thereby achieving peak shaving and valley filling of the power load, maximizing the local consumption of renewable energy, and significantly reducing carbon emissions [9].

Generally, TES is classified into three types: Sensible Heat Storage (SHS) [10], Latent Heat Storage (LHS) [11] and Thermochemical Heat Storage (TCHS) [12]. LHS utilizes the huge latent heat absorbed or released by PCM [13] during the solid–liquid or solid–solid phase change process for energy storage [14]. Its energy storage density is usually 5 to 10 times that of SHS, which can significantly reduce the device volume. Moreover, it has the characteristic of approximate constant temperature during the charging and discharging processes, which fits well with the temperature stability requirements of building heating and cooling [15]. The overall efficacy of any LHS system is intrinsically tied to the heat transfer characteristics of its internal exchanger [16]. To accelerate the thermal energy storage and recovery cycles, continuous efforts have been made to innovate and optimize the physical configurations of these heat transfer units.

Latent heat exchangers are mainly classified based on the contact method between PCM and heat transfer fluid (HTF) as well as their geometric structure. Although the direct contact type has extremely high heat exchange efficiency, it is less used in engineering due to the difficulties of material miscibility and separation [17]. Currently, the mainstream indirect contact type transfers heat through a solid wall. Indirect contact latent heat exchangers are mainly divided into Shell-and-Tube [18], Plate [19], Packed Bed [20], and Heat Pipe Integrated [21]. Salman et al. [22] point out that research introducing new passive technologies and multi-pass plate heat exchanger configurations is still limited compared to parameterized and 1-1 channel designs. Hussam et al. [23] adopted a novel designed shell spiral tube converter to improve converter efficiency, concluding that a water-MgO−Al_2_O_3_ nano-hybrid with a volume concentration of 0.5% is recommended to maximize the effectiveness of the shell-and-tube heat exchanger. Gopal et al. [24] successfully evaluated the thermal performance of shell-and-tube and tubular heat exchangers, using water and paraffin PCM as HTF and energy storage media.

The shell-and-tube heat exchanger has become the preferred form in building heat pump systems due to its advantages of high pressure resistance, robust structure, and ease of integrating enhanced heat transfer technologies (such as finned tube and corrugated tube) [25]. Fang et al. [26] point out the limitations of the traditional double-pipe heat storage system, finding that the thickening of the solidification layer in the inner tube leads to a significant increase in thermal resistance, limiting the effective heat storage ratio. To overcome this bottleneck, improving the thermal conductivity of PCM, optimizing the shell-and-tube structure, and increasing the heat transfer area have become key strategies. Mahdi et al. [27] showed that optimizing the geometric configuration of PCM (such as spherical structure) can improve the thermal response speed by about 30%. Li et al. [28] further found that compared to the aspect ratio, increasing the specific surface area of the heat exchanger plays a more significant and even dominant role in shortening the phase change time and improving the charging and discharging rates. Replacing conventional straight pipes with corrugated geometries introduces significant fluid flow disturbances and expands the available surface area for conduction. This dual enhancement mechanism has been robustly demonstrated to accelerate the phase-change transition and elevate overall thermal efficiency [29,30]. Experiments by Agrawal et al. [31] showed that a system with corrugated annular fins is superior to a finless system in terms of energy storage density and energy efficiency. El Mghari et al. [32] and Yang et al. [33] also confirmed through numerical simulation and experiments, respectively, that reasonably arranging the fin structure can not only increase the storage capacity of the device but also effectively accelerate the phase change process, achieving rapid charging and discharging.

Although finned and corrugated structures have been widely investigated, systematic comparisons of their enhancement mechanisms (extended surface area vs. induced turbulence) within a unified non-pressurized LHS framework are surprisingly scarce. Furthermore, most studies are limited to small-scale modules and lack full-cycle experimental validation coupled with actual ASHP systems. To address these gaps, this paper experimentally and numerically evaluates a novel NP-LHS device. By systematically comparing the transient charging/discharging behaviors of bare, corrugated, and finned tubes, this study aims to provide robust engineering guidelines for optimizing residential peak-shaving heating systems. Regarding the aforementioned issues, this paper proposes a novel Non-Pressurized Shell-and-Tube Latent Heat Storage (NP-LHS) system. Combining CFD numerical simulation and full-scale experimental research, the internal tube bundle spacing of the device is optimized, and the thermal performance differences between two typical enhanced heat-transfer structures, finned tube and corrugated tube, are systematically compared in a complete heat storage-release cycle. The aim is to provide a reliable theoretical basis and experimental support for the structural selection and optimization design of LHS devices in building ASHP. The main contributions of this paper include:

(1) A novel NP-LHS is proposed and verified. The device adopts a non-pressurized structural design, balancing operational safety and engineering implementability, and provides a feasible solution for the flexible replacement of PCM and the integrated application of building ASHP. (2) A systematic comparison of different enhanced heat transfer mechanisms is conducted under a unified device structure and operating conditions. Through the method of combining CFD numerical simulation and experiments, a quantitative comparative analysis is performed on the finned tube structure (dominated by extended surface area) and the corrugated tube structure (dominated by induced fluid disturbance) in a complete heat storage–release cycle, clarifying the performance differences and applicable characteristics of different tube types during heat storage and release processes. (3) Full-cycle experimental evaluation of the LHS device in an actual ASHP is realized. The proposed energy storage device is integrated into a real building ASHP to systematically evaluate its heat storage and release behavior under dynamic conditions and its ability to regulate indoor thermal comfort, providing an experimental basis for device selection and optimization in engineering applications.

**Figure 1:**
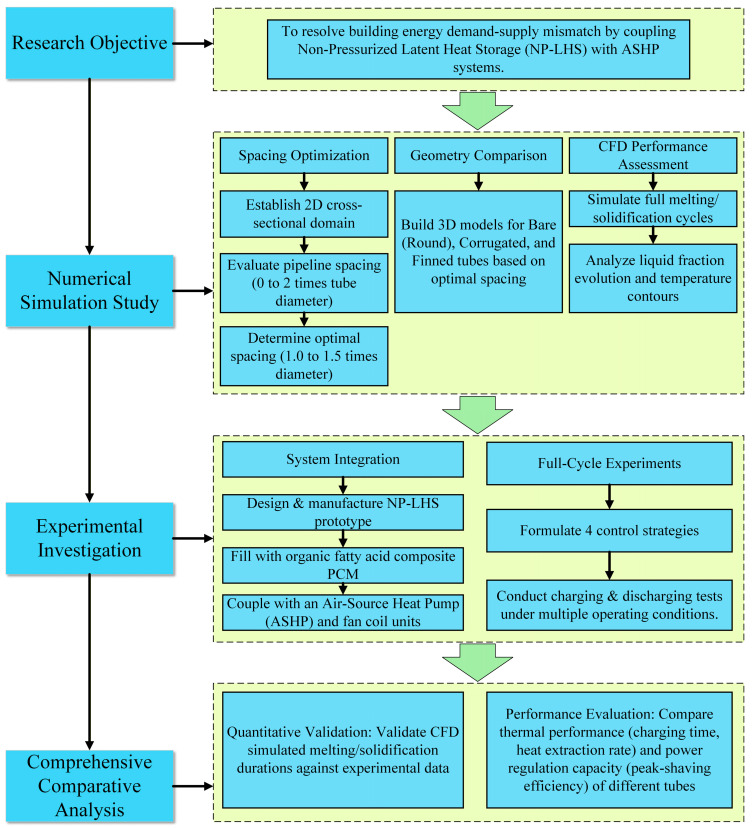
Overall research framework and flowchart of the numerical and experimental studies.

To provide a clear understanding of the comprehensive methodology adopted in this paper—integrating both numerical simulation and experimental verification across different tube geometries (smooth, corrugated, and finned tubes)—the overall research flowchart of the study is presented in Figure 1.

## 2. Principle of Novel Non-Pressurized Shell-and-Tube Latent Heat Storage (NP-LHS)

### 2.1. Structure of Latent Heat Storage (LHS) Device

**Figure 2:**
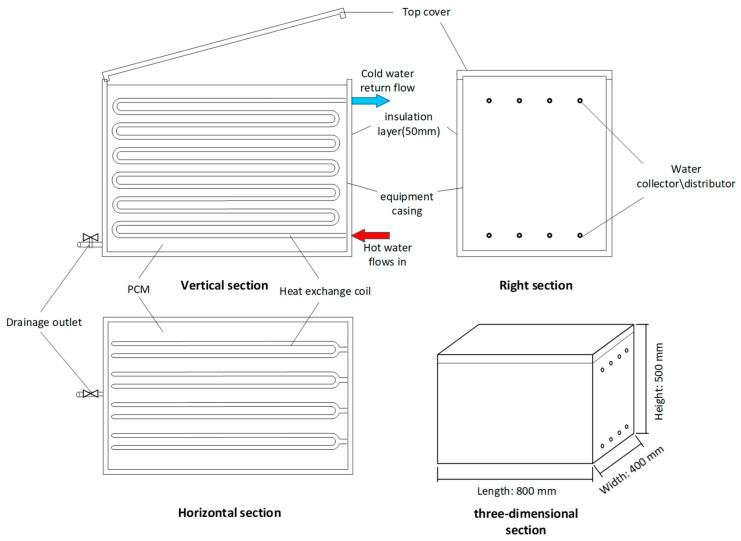
Schematic diagram of the structure of novel non-pressurized shell-and-tube latent heat storage (NP-LHS).

Based on the advantages of the shell-and-tube latent heat exchanger, such as a simple structure and strong heat exchange capacity, the basic structure of the Latent Heat Storage (LHS) device is proposed (Figure 2), adopting the form where the heat transfer fluid (HTF) flows inside the tube and the phase change material (PCM) is arranged outside the tube. Specifically, the LHS device adopts a cubic structure externally, which is convenient for combination with building spaces. Constructed from 304 stainless-steel paneling, the storage module features a non-hermetically sealed architecture to ensure operational versatility. The inclusion of an accessible top lid simplifies the initial injection of the phase-change medium, while a dedicated drainage port at the base allows for the rapid evacuation of the liquid PCM. This specific openable configuration provides the critical flexibility required to swap out thermal storage materials with varying melting points, dynamically adapting the system to distinct seasonal or operational heating loads. To minimize thermal leakage, the steel tank is insulated and shielded with a layer of polished aluminum foil. One side of the vessel features upper and lower ports that link the external manifold to the internal heat exchange coils.

Crucially, the NP-LHS employs a strict hydraulic decoupling mechanism between the pressurized heat pump system and the energy storage substance. The HTF from the ASHP flows exclusively inside the robust stainless-steel coil bundles, which are designed to withstand standard system operating pressures (e.g., 0.15–0.3 MPa). Conversely, the term “non-pressurized” applies solely to the outer cubic shell containing the PCM. This outer tank remains at atmospheric pressure, bearing only the hydrostatic weight of the liquid PCM. This decoupled design eliminates the necessity for manufacturing a heavy-duty, expensive pressure vessel for the bulk storage tank, significantly enhancing operational safety.

To provide a comprehensive understanding of the storage device, its specific geometry and dimensions are explicitly defined. The external dimensions of this NP-LHS housing are: length 800 mm × width 400 mm × height 500 mm. To minimize natural heat dissipation, the outer shell is wrapped with a 50 mm thick polyurethane insulation layer covered by polished aluminum foil.

The LHS device adopts an openable structure, which facilitates the replacement of PCM with different phase-change temperatures inside the device when the system operating temperature changes. The LHS device does not bear system pressure, which reduces the manufacturing difficulty, lowers the manufacturing cost, and improves the stability of operation.

### 2.2. Selection of PCM

LHS inherently capitalizes on the isothermal energy absorption of PCM during phase transitions, supplemented by minor sensible heat contributions. For integration into building ASHP networks, solid–liquid phase-change materials are highly preferred due to their substantial volumetric energy density and minimal thermal expansion. Consequently, the optimal selection of a thermal storage medium for residential applications is dictated by stringent criteria: the material must exhibit a phase-transition temperature precisely aligned with the heat pump’s operational window while simultaneously ensuring high latent capacity, chemical stability, non-toxicity, and economic viability.

**Figure 3:**
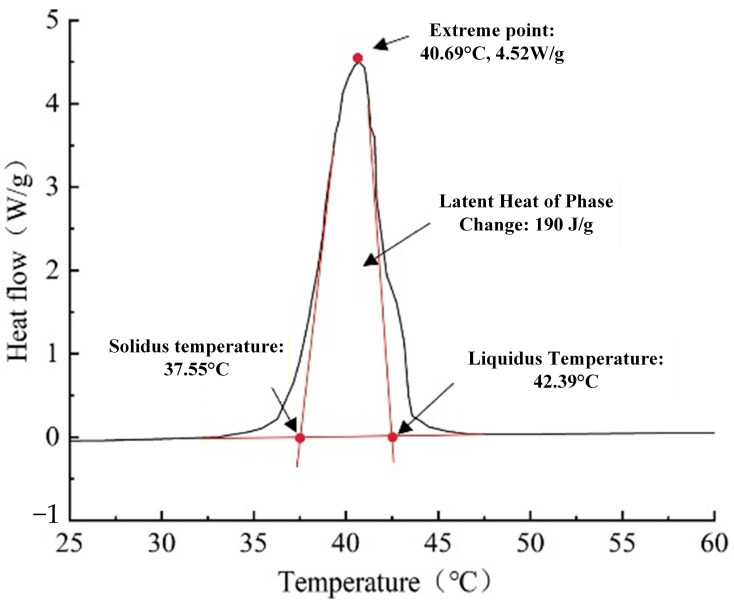
DSC curve of the selected phase change materials (PCMs). Adapted with permission from Ref. [36]. Copyright 2026.

Currently, PCM used for LHS devices mainly includes paraffin, water, fatty alcohols, fatty acids, and composite PCM. In typical residential ASHP systems, the supply water temperatures for heating usually range from 32 °C to 45 °C (occasionally reaching a maximum of 50 °C under peak loads), while the return water temperatures to the ASHP are significantly lower, typically between 20 °C and 30 °C. Considering these specific operational characteristics, the PCM selected for the LHS must possess a melting temperature precisely within this effective heat transfer window. Consequently, a type of organic fatty acid composite phase change material was selected, with its phase change peak temperature being 40.69 °C. Organic fatty acids are highly recommended for low- and medium-temperature thermal energy storage systems due to their high chemical stability, non-corrosiveness, and suitable phase change temperatures [34,35]. This PCM exhibits a solid–liquid phase transition within a narrow temperature range, with an onset melting temperature of 37.55 °C and a peak temperature of 40.69 °C, as determined by Differential Scanning Calorimetry (DSC). Its main components are glyceryl tristearate, glyceryl tripalmitate, and glyceryl trimyristate. The cost of the PCM used in this study is approximately 4.20 USD/kg. It is non-toxic and harmless to the human body, with a phase change energy storage density of 190 kJ/kg. As depicted in the DSC thermogram (Figure 3), the composite PCM exhibits excellent thermal reversibility, with its melting and cooling (solidification) curves being nearly identical. Consequently, the phase-change behavior is characterized using rigorous thermodynamic terminology: the solidus temperature (where melting begins or solidification ends) is 37.55 °C, and the liquidus temperature (where melting ends or solidification begins) is 42.39 °C, with a peak phase-change temperature at 40.69 °C. This highly coincident heating and cooling behavior indicates that the selected organic PCM possesses a negligible degree of subcooling. This is a critical material advantage for the discharging (heat release) phase of the ASHP system, as it ensures that the PCM can initiate crystallization and release its latent heat of phase change (190 kJ/kg) promptly and stably as soon as the temperature drops below the liquidus point, thereby maintaining a consistent heat supply to the fan coil units.

The reliable execution of the ensuing CFD models relies intrinsically on the precise characterization of the energy storage medium. Consequently, the comprehensive phase-dependent thermophysical parameters governing the thermal response of the chosen organic composite are detailed in Table 1.

### 2.3. Heat Transfer Analysis of LHS Device

Analyzing the charging and discharging cycles helps explain how heat moves and energy accumulates within the module.

(1) Initially, the PCM is entirely solid and stays below its melting temperature. In this stage, energy from the coils spreads into the surrounding material through simple conduction.

(2) As heat storage proceeds and as the area near the tubes gets hotter and passes the melting point, the PCM starts to liquefy. This creates a molten layer around the pipes while the rest of the material remains solid. This represents the shift from sensible heating to latent energy storage. For plain and corrugated tubes, as the liquid PCM layer thickens, the conductive thermal resistance increases, and natural convection begins to dominate. However, for the finned tube configuration, the extended metal surfaces provide continuous, highly conductive pathways, significantly mitigating the decline in the heat conduction effect even in the liquid phase.

(3) As heat storage continues, the expanding liquid layer dictates that natural convection governs the internal heat distribution, as the solid PCM fraction continuously diminishes. Once full liquefaction is achieved, the sensible heating of the molten PCM dominates until its temperature converges with that of the inlet HTF.

Throughout this entire sequence, the net energy transfer is strictly directed from the actively pumped HTF into the surrounding phase-change matrix. To model the heat transfer along the flow direction, the heat exchange tube is discretized into n differential segments along its longitudinal axis, with the *i*-th segment representing a local control volume. The thermal energy *Q*_1_,*_i_*, transferred from the HTF to the PCM domain within the *i*-th differential segment during charging, is evaluated using Equation (1).

(1) $$Q_{1,i}=k_{1}A_{1}(T_{1,i}-T_{p,i})\tau$$

where *k*_1_ is the heat transfer coefficient based on the inner wall surface of the coil, W/(m^2^·K) (Equation (2)); *A*_1_ is the area of the wall surface, m^2^; *T*_1,_*_i_* is the temperature of the HTF in the *i*-th differential segment, °C; *T_p_*_,_*_i_* is the temperature of the PCM in contact with the outer wall of the heat exchange coil, °C; τ is the heat exchange duration, s.

(2) $$k_{1}=\frac{1}{\frac{1}{h_{1}}+\frac{r_{i}ln(r_{o}/r_{i})}{\lambda }+\frac{r_{i}}{r_{o}h_{2}}}$$

where *h*_1_ is the inner wall heat transfer coefficient, W/(m^2^·K); *r_i_* and *r_o_* are the inner and outer radii of the tube, m; λ is the tube wall thermal conductivity, W/(m·K); *h*_2_ is the outer wall heat transfer coefficient, W/(m^2^·K). It should be noted that *h*_2_ is highly transient and variable throughout the melting and solidification processes, as it is fundamentally governed by the evolving Nusselt number associated with the buoyancy-driven natural convection in the liquid PCM. Equations (1) and (2) are presented here strictly for the purpose of qualitative heat transfer analysis, to elucidate the governing thermal resistances. The actual quantitative evaluation of the complex, transient phase-change behavior relies entirely on the robust full-scale experimental measurements and the rigorous CFD numerical simulations.

Then, the total heat exchange amount between the HTF and the PCM is calculated by Equation (3).

(3) $$Q_{1}=\sum_{i=1}^{n}Q_{1,i}$$

where *Q*_1_ is the total heat exchange amount between the HTF and the PCM, J.

The heat exchange amount between the PCM of different phase states during the heat storage and release processes is denoted as *Q*_2_. The heat exchanged inside liquid and solid PCM is denoted as *Q*_3_ and *Q*_4_, respectively. The heat absorbed by PCM undergoing phase change during the heat transfer process is denoted as *Q*_5_. Each part of the heat can be calculated by Equations (4)–(7).

(4) $$Q_{2}=Q_{4}+Q_{5}$$

(5) $$Q_{3}=\sum_{t=1}^{\infty }Q_{3,t}=\sum_{t=1}^{\infty }c_{l}m_{l,t-1}(T_{l,t}-T_{l,t-1})$$

(6) $$Q_{4}=\sum_{t=1}^{\infty }Q_{4,t}=\sum_{t=1}^{\infty }c_{s}m_{s,t-1}(T_{s,t}-T_{s,t-1})$$

(7) $$Q_{5}=\sum_{t=1}^{\infty }Q_{5,t}=\sum_{t=1}^{\infty }h(m_{s,t-1}-m_{s,t})$$

In the above expressions, *Q*_3,*t*_, *Q*_4,*t*_ and *Q*_5,*t*_ denote the sensible heat absorbed by the liquid phase, the sensible heat absorbed by the solid phase, and the latent heat absorbed during the phase transition at a given time step *t*, respectively. The variables *m* and *T* represent the transient mass and temperature of the respective phases, while *c* and *h* denote the specific heat capacity and the latent heat of fusion. A comprehensive list of all symbols, subscripts (e.g., *l* for liquid, *s* for solid), and their corresponding units is provided in the Nomenclature section at the end of this paper.

Heat release is driven by the temperature difference between the colder HTF and the warm PCM. This discharging process involves three stages:

(1) At the initial stage of heat release, the liquid PCM is above its melting point. Energy moves from the liquid to the tube walls via convection and then conducts into the HTF.

(2) As heat release proceeds, the PCM near the tubes reaches its phase-change temperature and starts to freeze. A solid layer forms on the pipes, releasing latent heat.

(3) As heat release continues, the solid layer grows thicker. This solid PCM has low conductivity and acts as an insulator, increasing thermal resistance. As a result, the heat release rate drops until it can no longer meet the system’s load.

Throughout this cycle, heat always flows from the PCM to the HTF. The math uses the same equations as the charging phase, where a negative heat flux result simply indicates the system is discharging.

## 3. Simulation Optimization of LHS Device

Given that the embedded coil acts as the exclusive thermal interface bridging the circulating HTF and the static PCM, the macroscopic charging and recovery capacities of the storage tank rely entirely on the efficacy of this heat exchange boundary. Therefore, optimizing the structural and dimensional parameters of the internal pipeline is paramount for maximizing the operational effectiveness of the device. A three-dimensional numerical simulation of the heat storage unit was performed using the Solidification/Melting model in ANSYS Fluent 2022. The phase change process involves highly non-linear, multi-physics heat transfer mechanisms, including sensible heat effects and fluid–solid thermal interactions. The geometric model was constructed in Design Modeler. The phase-change behaviors (melting and solidification) and heat transfer characteristics were simulated using the finite volume method, with the pressure-velocity coupling resolved by the SIMPLE algorithm.

### 3.1. Simulation Study on the Influence of Pipeline Spacing on the Heat Storage and Release Effect of the Storage Device

**Figure 4:**
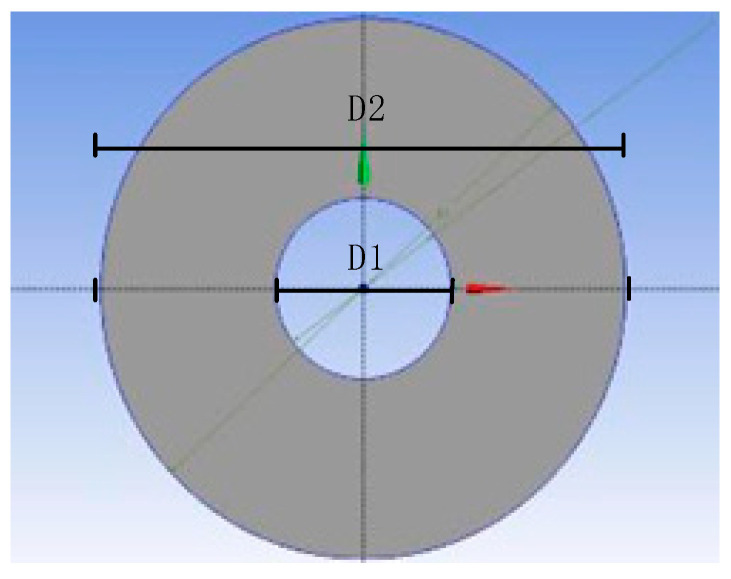
Geometric structure of the differential simulation object section.

The pipeline spacing inside the device is one of the key factors affecting the heat storage and release efficiency of this storage device. In the simulation study, a single pipeline and the PCM within a specified range are longitudinally divided into n parts of differential elements. One of the differential elements is selected as the research object to establish the geometric structure of the longitudinal section of the storage device. The established geometric structure is based on the longitudinal section of the pipeline and is a circular ring bounded by the circular boundary of the PCM (Figure 4). In the figure, D1 is the diameter of the HTF pipeline (20 mm); D2 is the outer boundary diameter of the PCM domain (60 mm); the part between D1 and D2 is the PCM used in the experiment.

It should be noted that throughout this manuscript, Celsius (°C) is primarily used to describe macroscopic experimental operating conditions and indoor environmental parameters, in accordance with standard HVAC engineering practices. Conversely, Kelvin (K) is exclusively utilized within the computational fluid dynamics (CFD) modeling sections to align with the absolute temperature scale natively required by the solver’s internal thermodynamic and Boussinesq approximation calculations (T (K) = T (°C) + 273.15).

The computational domain was initialized with the PCM entirely in the solid phase at a uniform baseline temperature of 313 K. During the simulated charging cycle, the exterior surfaces of the heat transfer tubes—serving as the direct thermal interface to the PCM—were maintained at a constant 323 K. To analyze the transient thermal response, both the instantaneous temperature fields and the evolving liquid fraction contours were continuously recorded. The liquid fraction is calculated using the enthalpy-porosity formulation in ANSYS Fluent, where the liquid fraction *β* is defined as a linear function of temperature within the mushy zone, representing the ratio of the liquid volume to the total cell volume. The complex melting progression was resolved using the enthalpy-porosity formulation, a technique that circumvents the explicit tracking of the moving solid–liquid boundary. Instead, it computes a localized liquid fraction (*β*) for each control volume, providing a continuous, implicit representation of the phase-change front. A stringent convergence criterion was enforced, requiring all scaled residuals to fall below 10^−6^ at each time step.

To ensure the numerical accuracy and computational efficiency of the CFD model, rigorous mesh independence and time-step independence tests were conducted prior to the main simulations.

**Figure 5:**
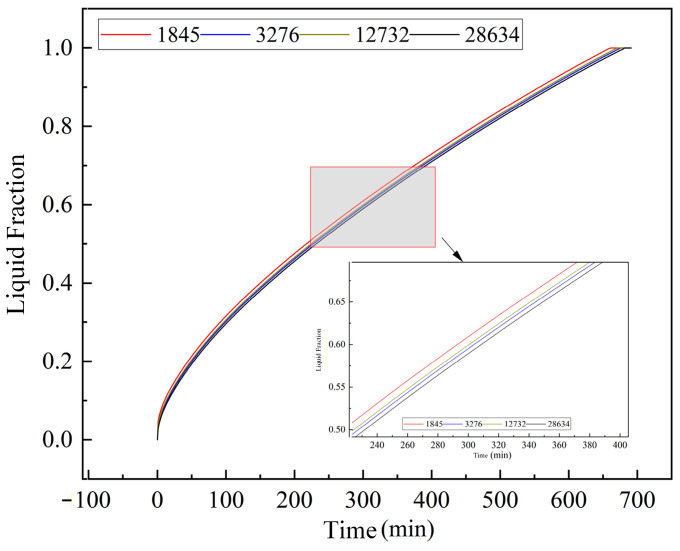
Variation of liquid fraction with different grid numbers.

Taking the 2D cross-sectional model as a benchmark, four different grid resolutions were evaluated: 1845, 3276, 12,732, and 28,634 cells. The transient variation of the PCM liquid fraction was monitored for each grid size (as shown in Figure 5). The results indicate that the liquid fraction curves for the 3276, 12,732, and 28,634 cell meshes are virtually identical, with a maximum relative deviation of less than 1.5%. Therefore, to balance calculation precision and computational cost, a mesh density equivalent to the 3276-cell configuration was selected as the standard for subsequent simulations. Based on this standard, the final optimized 3D grids for the full-scale bare tube, corrugated tube, and finned tube models were established at 674,103, 6,948,376, and 3,014,781 cells, respectively.

**Figure 6:**
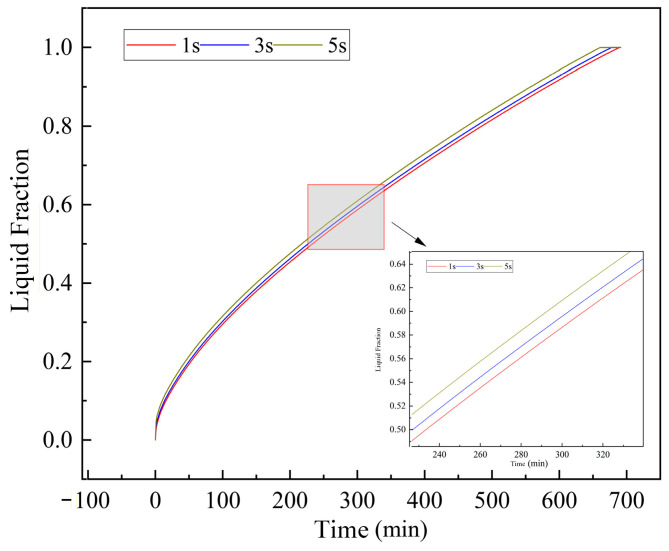
Liquid fraction curves under different time steps.

Since the melting and solidification of PCM are highly transient processes, selecting an appropriate time step is crucial. Time steps of 1 s, 3 s, and 5 s were tested using the chosen mesh (as shown in Figure 6). The liquid fraction evolution demonstrated negligible differences between the 3 s and 5 s time steps. Consequently, a time step of 5 s was adopted for all simulations, ensuring accurate temporal resolution while significantly reducing the total computational time. The convergence criterion was set to a residual of less than 10^−6^ for the energy equation and 10^−4^ for the continuity and momentum equations.

**Figure 7:**
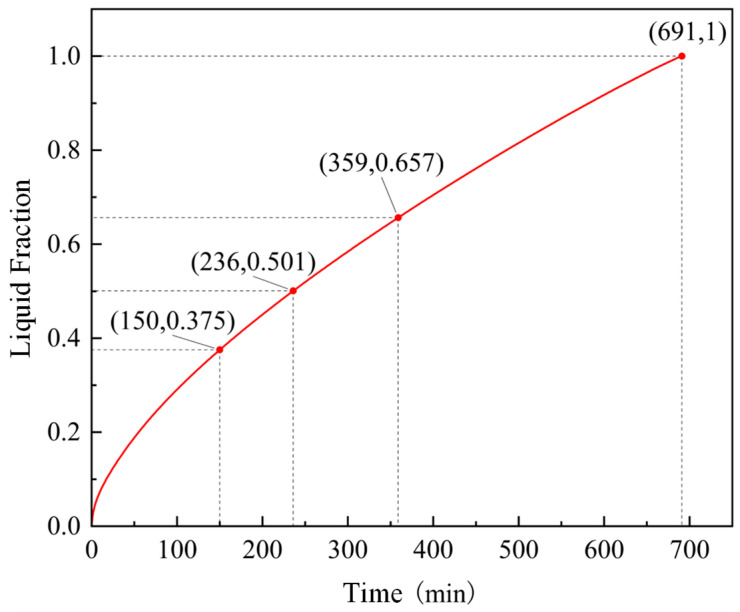
Simulation curve of PCM liquid fraction variation during heat storage process.

According to the verified time step and grid, the curve of the liquid fraction of PCM changing with time during the heat storage process is shown in Figure 7. In the initial stage of heat storage, the growth rate of the PCM liquid fraction is relatively large. As heat storage proceeds, the growth rate of the liquid fraction gradually decreases. Specifically, when the heat storage duration is 236 min, the PCM liquid fraction reaches 0.501. When the heat storage duration is 691 min, the PCM is completely liquefied. The second half of the PCM liquefaction duration increased by 92.8% compared to the first half. It must be emphasized that a charging duration of 691 min (over 11 h) is practically unacceptable for daily peak-shaving operations in building heating systems, where the available off-peak electricity window typically lasts only 6 to 8 h overnight. This excessively long duration fundamentally exposes the critical limitation of utilizing low-thermal-conductivity organic PCMs with conventional bare tube heat exchangers. The rapidly thickening layer of liquid PCM acts as a severe thermal insulator, drastically slowing down the melting rate in the later stages. This unacceptable performance of the bare tube configuration provides the core justification and imperative for introducing advanced heat transfer enhancement structures—specifically, the corrugated and finned tubes investigated in the subsequent sections of this study—to reduce the charging time to a practical engineering timeframe.

**Figure 8:**
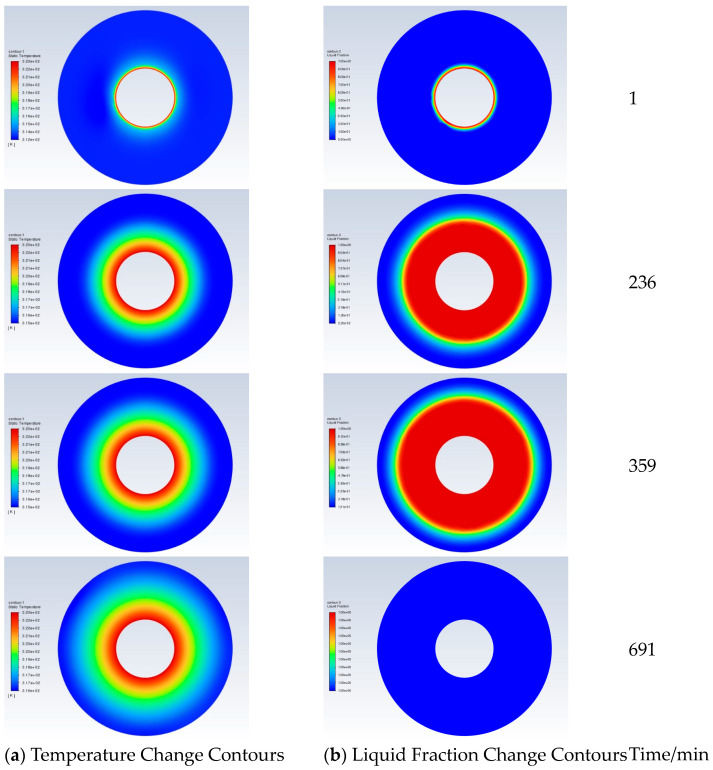
Section liquid fraction and temperature contours in each time period of heat storage.

The liquid phase change contour and temperature change contour of PCM in each time period of heat storage are shown in Figure 8a,b, respectively. At 1 min of heat storage, the temperature contour distribution of the pipeline section is approximately elliptical, and the temperature in the vertical direction is higher than that in the horizontal direction. As heat storage proceeds, the temperature contour distribution tends to be circular. When the heat storage duration is 150 min, the solid–liquid interface diameter of the PCM reaches one times the tube diameter. When the heat storage duration is 236 min, the solid–liquid interface of the PCM reaches 1.23 times the tube diameter. When the heat storage duration is 359 min, the solid–liquid interface of the PCM reaches 1.5 times the tube diameter. At the end of heat storage, the solid–liquid interface of the PCM reaches two times the tube spacing.

Combined with the liquid fraction curve, it can be seen that after the solid–liquid interface reaches 1.23 times the tube spacing, the growth rate of the liquid fraction of the pipeline section slows down. After the pipeline spacing reaches 1.5 times the tube spacing, the growth rate of the PCM liquefaction rate is small, and the heat storage duration is long. Therefore, during the heat storage process, when the internal pipeline spacing of the device is between 1 time and 1.5 times the tube diameter, the storage device can ensure a relatively reasonable heat storage duration. This is consistent with the heat storage and release experimental laws of the corrugated tube storage device of our research group [37].

### 3.2. Simulation Study on the Influence of Pipeline Type on the Heat Storage and Release Effect of the Storage Device

Three common pipeline types, round tube, corrugated tube, and finned tube, are selected for simulation research. Among them, the round tube is the basic tube type of the pipeline and serves as a benchmark for comparison; the corrugated tube and finned tube achieve the purpose of enhanced heat transfer by increasing the heat transfer area on the basis of the round tube. The applicability of the three in the LHS device is analyzed through simulation.

#### 3.2.1. Simulation Study on Heat Storage and Release of Round Tube Storage Device

Based on the study of pipeline spacing, 1.25 times the tube spacing is adopted in the simulation. Considering that this storage device has high symmetry, the impact on the results after performing symmetrical scale-down research is small, so scale-down research is adopted for the storage device. The geometric dimensions of the scaled-down round tube storage device are 200 mm length, 200 mm width, and 125 mm height. The storage device adopts four columns of round tubes internally. The round tube columns inside the device are evenly distributed horizontally. The diameter of the round tube is 25 mm. The volume proportion of PCM inside the round tube storage device is 77%. Each column of round tubes has three rows of horizontal tubes. The adopted pipeline bending diameter can ensure that the horizontal tubes are evenly distributed inside the device.

**Figure 9:**
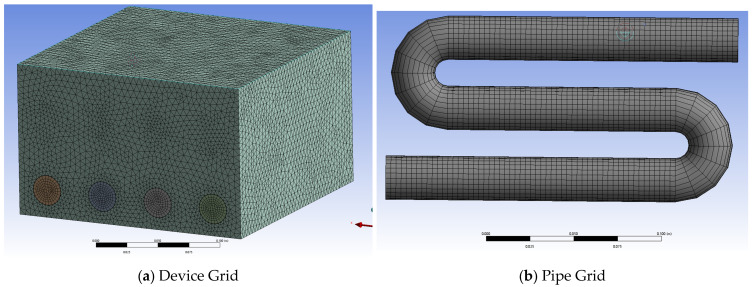
Grid meshing of round tube storage device.

After repeated debugging and comparison in calculation, the optimal time step set for the round tube storage device is determined to be 5 s, and the grid number is 674,103 (Figure 9).

In the heat storage simulation, the pipeline opening at the bottom of the device is the HTF inlet, defined as the velocity inlet, and the pipeline opening at the top of the device is the HTF outlet, defined as the outflow boundary. The inlet temperature remains unchanged at 323 K during the heat storage process. Simulations are performed for pipeline inlet flow velocities set to 0.05 m/s, 0.1 m/s, and 0.2 m/s, respectively. In the heat release simulation, the pipeline opening at the bottom of the device is the HTF outlet, defined as the outflow boundary, and the pipeline opening at the top of the device is the HTF inlet, defined as the velocity inlet. The HTF inlet flow velocity remains unchanged at 0.1 m/s during the heat release process.

**Figure 10:**
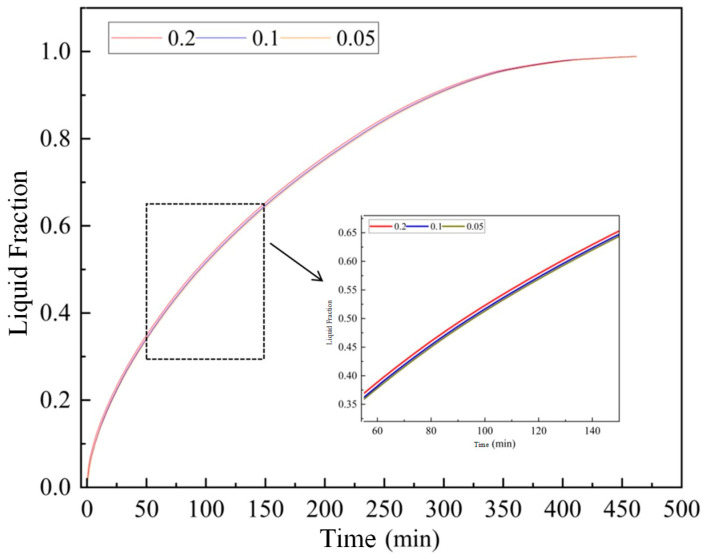
PCM liquid fraction curves under different inlet flow velocity conditions.

To precisely capture the transient thermal response of the plain tube storage unit during the charging and discharging cycles, the spatial evolution of the temperature and liquid fraction was continuously logged across both the central transverse plane and a representative longitudinal cross-section within the tube bundle. Focusing on the charging phase, Figure 10 illustrates the temporal variation of the volumetric melt fraction under varying inlet velocities of the HTF. A detailed analysis reveals a distinct, stage-dependent sensitivity to the flow rate. During the initial sensible heating and the final thermal saturation periods, augmenting the HTF velocity yields negligible acceleration in the overall liquefaction rate. Conversely, during the active, convection-dominated middle stage of the melting process, a pronounced positive correlation emerges: higher HTF inlet velocities noticeably enhance the macroscopic melting rate, resulting in a substantially greater liquid fraction at any given moment.

**Figure 11:**
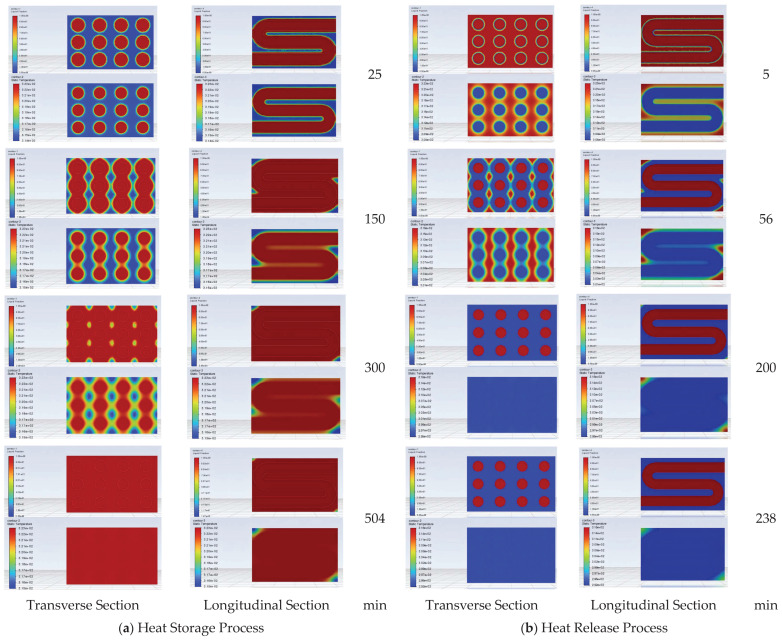
Contour changes of round tube storage device during heat release process.

The changes in temperature and liquid fraction of the transverse section and longitudinal section of the round tube storage device during the heat storage experimental process are shown in Figure 11a. Among them, the upper part of the contour at each moment is the liquid fraction contour, and the lower part is the temperature contour. From the transverse section contour, it can be known that in the initial stage of heat storage, the PCM inside the device mainly undergoes phase change near the pipeline. As heat storage proceeds, the liquid PCM between vertical pipelines connects together first; then, the PCM between horizontal pipelines undergoes liquefaction connection. From the longitudinal section contour, it can be known that as heat storage proceeds, the liquefaction time of the PCM at the corners of the storage device is later. For a long time in the late stage of heat storage, the PCM at the corners of the storage device still cannot undergo liquefaction.

The liquid fraction and temperature change contours of the transverse section and longitudinal section of the round tube storage device are shown in Figure 11b. The PCM around the pipeline solidifies first to release latent heat, while the PCM at the corners of the device solidifies last. When the distance between the liquid PCM and the pipeline is too far, the heat release rate slows down due to the low thermal conductivity of the PCM.

**Figure 12:**
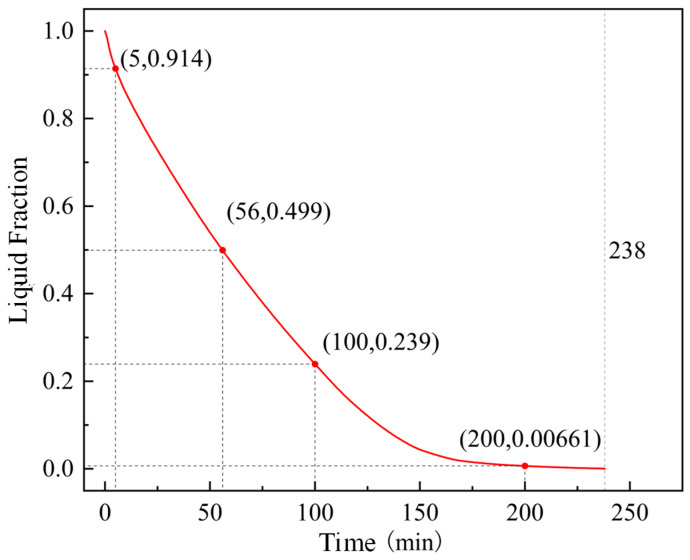
Liquid fraction curve of round tube storage device during heat release.

The curve of the liquid fraction of PCM inside the round tube storage device in the heat release simulation is shown in Figure 12. In the early stage of heat release, the liquid fraction inside the storage device decreases rapidly, while in the late stage of heat release, the decrease in the liquid fraction inside the storage device slows down. When the heat release duration is 56 min, the liquid fraction of the PCM inside the device is already lower than 50%, while it takes 182 min for the liquid fraction inside the storage device to decrease from 50% to close to 0. This indicates that as heat release proceeds, the latent heat inside the storage device becomes more difficult to release.

#### 3.2.2. Simulation Study on Heat Storage and Release of Corrugated Tube and Finned Tube Storage Devices

**Figure 13:**
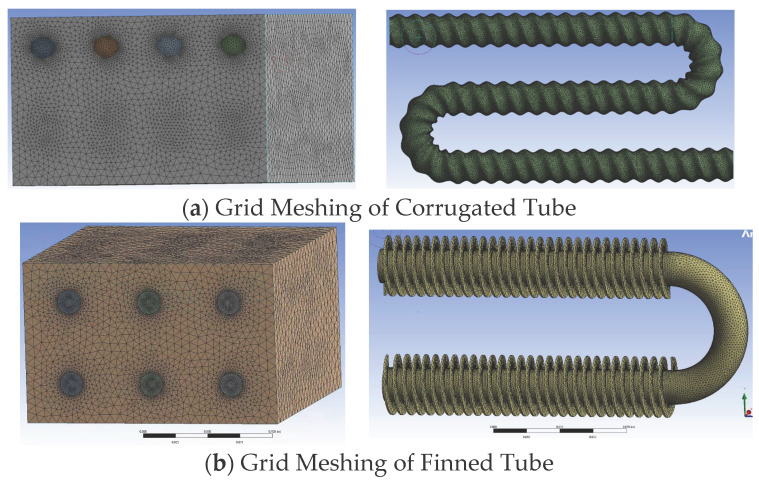
Grid meshing of corrugated tube and finned tube storage devices.

The corrugated tube storage device is subjected to the same scale-down treatment as the round tube storage device. The external geometric dimensions of the scaled-down model of the corrugated tube storage device are the same as those of the bare tube. Internally, the large diameter of the corrugated tube used is 20 mm, the small diameter is 16 mm, and a total of four rows of pipelines are set. The volume proportion of PCM inside the device is 88.2%. The pipelines are evenly distributed horizontally inside the device. Each row of pipelines is set with three horizontal tubes. The adopted pipeline bending diameter can ensure that the vertical pipelines are evenly distributed inside the device. After repeated debugging and comparison in calculation, the optimal time step determined for the corrugated tube storage device is 5 s, and the grid number is 6,948,376. The grid meshing is shown in Figure 13a.

The external geometric dimensions of the scaled-down model of the finned tube storage device are the same as those of the bare tube. The straight tube part is the finned tube, and the bent tube part is still the round tube. The geometric parameters of the finned tube used are: tube diameter 18 mm, fin height 7 mm, fin thickness 1 mm, and fin spacing 5 mm. The volume of PCM inside the finned tube storage device is 92.5%. The adopted pipeline bending diameter can ensure that the finned tube inside the device is evenly distributed in the horizontal and vertical directions. After repeated debugging and comparison in calculation, the optimal time step determined for the finned tube storage device simulation is 5 s, and the grid number is 3,014,781. The grid meshing is shown in Figure 13b.

For the corrugated tube storage device and the finned tube storage device, the simulation initial parameter settings for both heat storage and heat release working conditions are the same as those for the round tube storage device. This setting makes it easier to compare and analyze the heat storage and release performance parameters of the three through simulation results. In the low flow velocity range, the HTF inlet flow velocity has little effect on the heat storage duration of the storage device. Therefore, in the heat storage working conditions of corrugated tube and finned tube, only the working condition with an inlet flow velocity of 0.1 m/s is selected for simulation.

**Figure 14:**
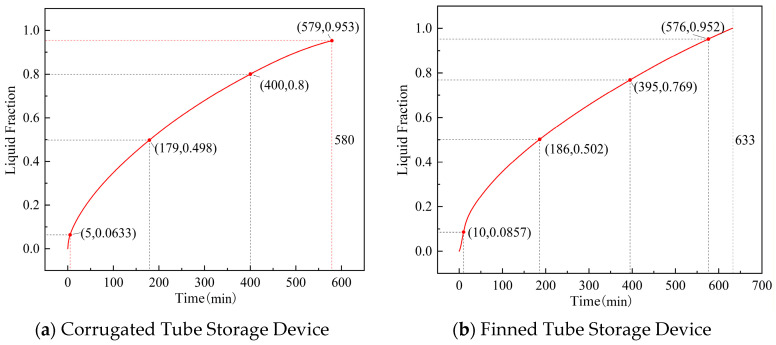
Liquid fraction curves of storage devices during heat storage.

The variation curves of the PCM liquid fraction of the corrugated tube and finned tube storage devices during the heat storage process are shown in Figure 14. During the heat storage process, the liquid fraction inside the corrugated tube storage device and the finned tube storage device reaches 50% at heat storage durations of 179 min and 186 min, respectively, which are relatively close in time. However, the finned tube storage device has a larger proportion of PCM inside, so the finned tube storage device has a larger energy storage density and a faster heat storage rate.

**Figure 15:**
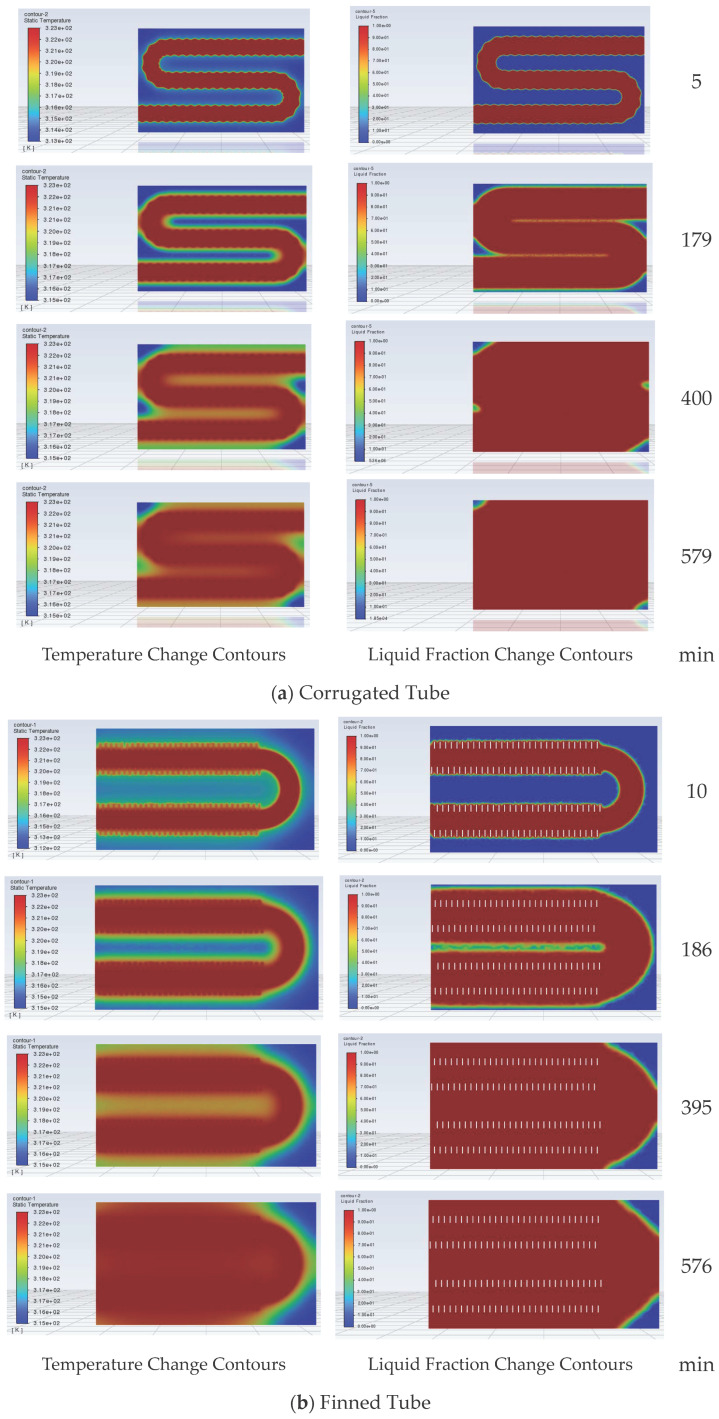
Heat storage process contours of corrugated tube and finned tube storage devices.

In the heat storage and heat release simulations of the corrugated tube and finned tube, the longitudinal section (parallel to the tube length direction) along the maximum tube diameter of the finned tube bundle can more intuitively reflect the influence laws of fin spacing and tube spacing on the temperature field and liquid fraction distribution. Therefore, the temperature and liquid fraction changes of the longitudinal section are selected as the main monitoring objects. The changes in liquid fraction at different moments of the heat storage process for the two tube types are shown in Figure 15a,b, respectively. It can be seen that for the corrugated tube, the temperature and liquid fraction change contours during its heat storage process are close to those of the round tube storage device. For the finned tube, although the phase change also occurs around the pipeline in the early stage of heat storage, the PCM between the fins undergoes phase change first. Since the bent pipe of the finned tube is far from the corners of the device, it is difficult for the PCM at the corners inside the device to melt.

**Figure 16:**
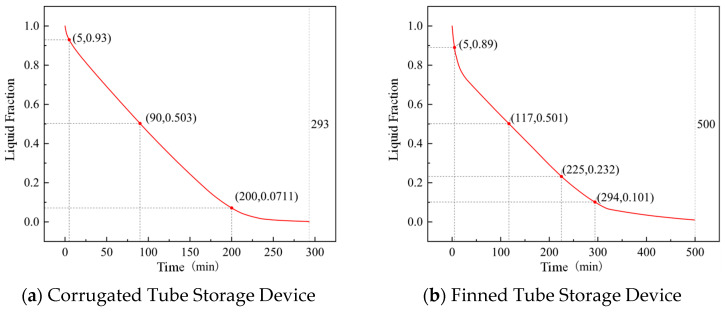
Liquid fraction curves of storage devices during heat release.

The liquid fraction curves of corrugated tube and finned tube storage devices during the heat release process are shown in Figure 16. During the heat release process, the rate of change of the liquid fraction of the PCM inside both devices gradually decreases. For the corrugated tube, when the heat release duration is 90 min, the liquid fraction inside the device is close to 50%, and the liquid fraction does not approach 0 until the heat release duration reaches 293 min. For the finned tube, when the heat release duration is 117 min, the liquid fraction inside the device is close to 50%, and the liquid fraction is 0 until the heat release duration is 500 min. It can be seen that for the finned tube storage device, the liquid fraction changes slowly in the late stage of heat release, significantly prolonging the heat release duration of the finned tube storage device. This is because the bent pipe inside the designed finned tube storage device is a circular bare tube, and the distance between the pipeline and the device corners is large, leading to a slowed heat release rate of the PCM inside the device in the late stage of heat release. Reducing the spacing between the device corners and the bare tube will accelerate the heat release of the finned tube storage device.

Even so, in the early stage of heat release, the difference in heat release rate between the corrugated tube storage device and the finned tube storage device is still small. This indicates that the finned tube storage device has greater room for improvement, and adopting the finned tube inside the LHS device can increase the heat release performance of the LHS device.

**Figure 17:**
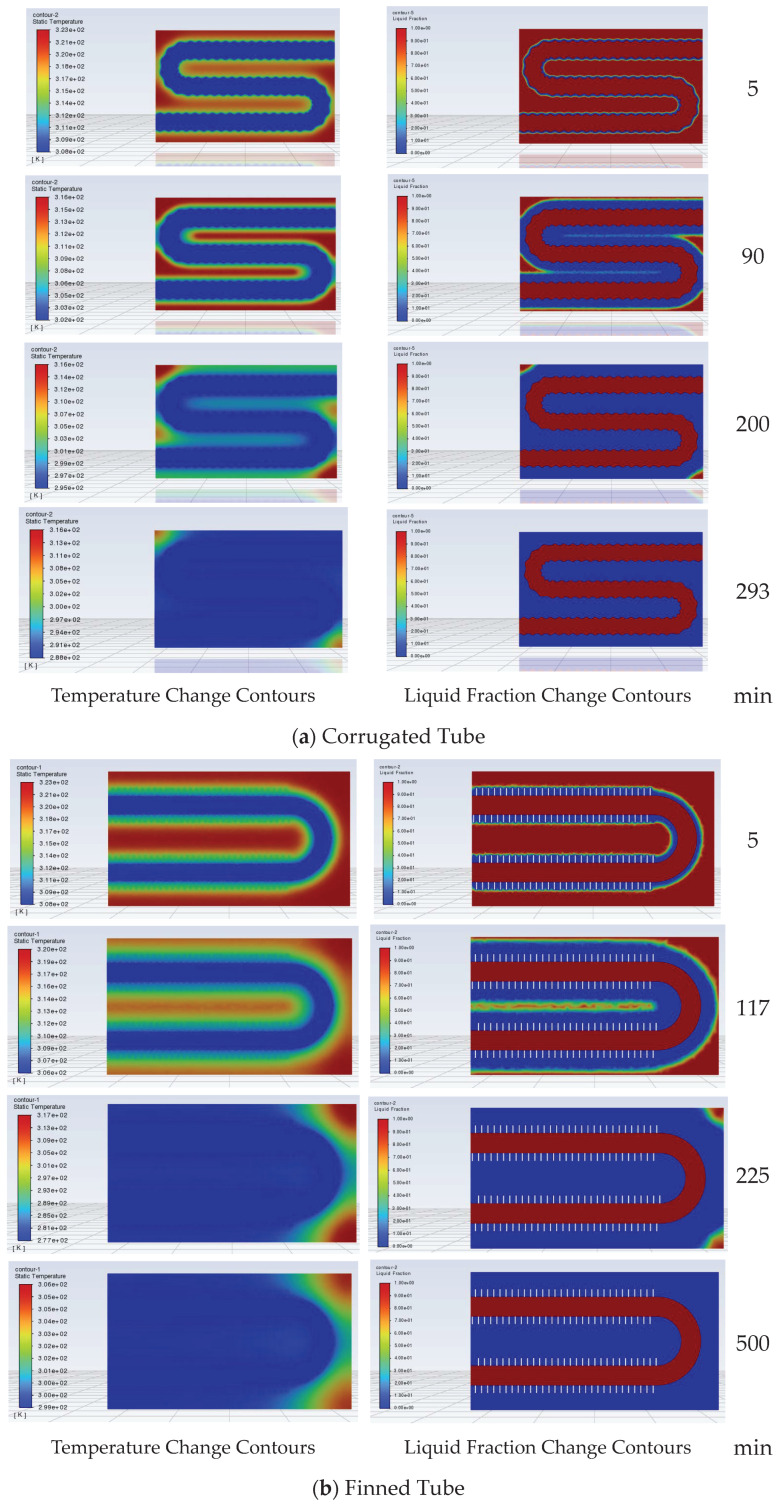
Heat release process contours of corrugated tube and finned tube storage devices.

The longitudinal section temperature and liquid fraction change contours of the corrugated tube and finned tube under heat release working conditions are shown in Figure 17a,b, respectively. During the heat release process, the PCM at the corners inside the corrugated tube storage device still undergoes phase change last to complete heat release. For the finned tube, in the initial stage of heat release, the PCM between the fins preferentially undergoes phase change. Afterwards, the phase change of the PCM gradually diffuses from around the pipeline to the outside, and the PCM at the corners of the device undergoes phase change last.

## 4. Experimental Investigation of the NP-LHS System

### 4.1. Experimental Setup and System Integration

#### 4.1.1. Principle of ASHP Equipped with NP-LHS

**Figure 18:**
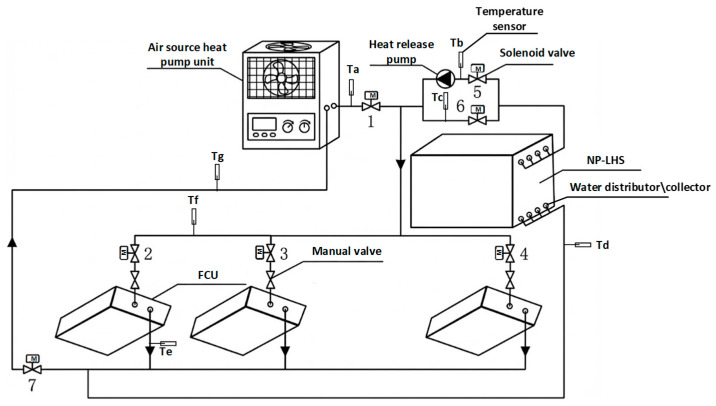
Principle of ASHP equipped with NP-LHS. Adapted with permission from Ref. [37]. Copyright 2026.

After the structural design of the LHS water tank is completed, it needs to be connected to the ASHP air-conditioning system. Traditional LHS water tanks require independent heat pump units, which makes the method complex, increases retrofitting costs due to the addition of units to the existing system, and hinders the promotion of the energy storage device. To this end, an LHS system was designed that can directly install the aforementioned LHS water tank onto a common ASHP air-conditioning system and equip it with auxiliary components such as solenoid valves to achieve switching between different operating modes. The system principle is shown in Figure 18.

A parallel connection was established between the fan coil units and the proposed NP-LHS module within the ASHP system. The connection point between the LHS device branch and the outlet side of the ASHP unit is divided into two branches: the “Heat Storage Branch” and the “Heat Release Branch”. The flow through these branches is switched by solenoid valves. The heat storage branch is connected to basic components such as temperature sensors, electromagnetic flowmeters, pressure transmitters, and manual regulating valves, which are used to monitor the situation during the device’s heat storage. In addition to the above basic components, the heat release branch is equipped with a circulation pump. In the energy release condition, it provides power for the circulation of the HTF (heating medium water) in the tube to supply heat to the terminal fan coil units.

The system operates under four distinct modes (Conventional, Independent Storage, Joint Operation, and Energy Release), governed by the coordinated opening and closing of solenoid valves, as summarized in Table 2.

#### 4.1.2. Experimental Platform and Equipment

The ASHP unit is equipped with three fan-coil-unit heat-release terminals, located in Room A, Room B, and Room C, respectively. The detailed parameters of the equipment are shown in Table 3.

**Figure 19:**
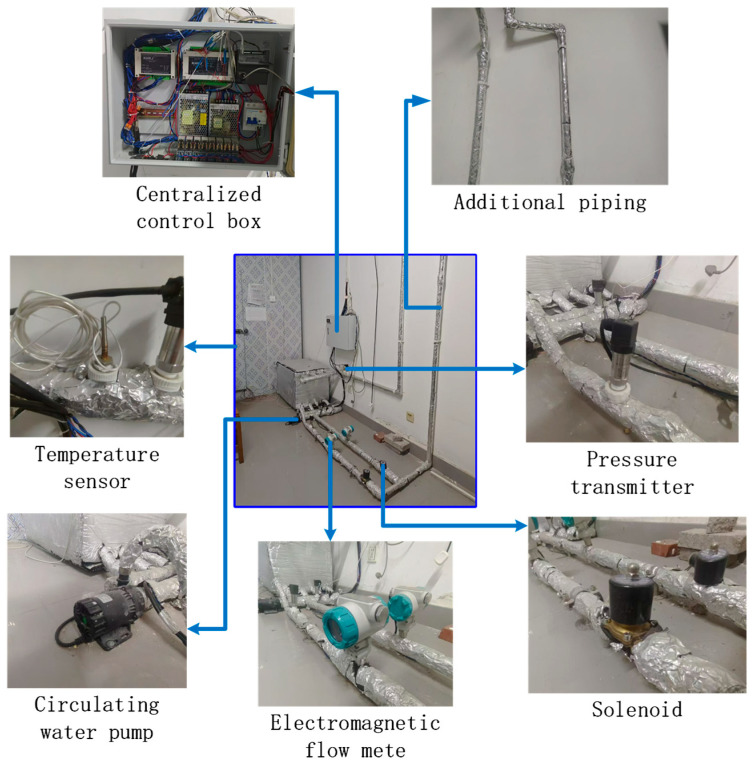
NP-LHS system monitoring component layout. Adapted with permission from Ref. [36]. Copyright 2026.

Key nodes of the ASHP system equipped with the NP-LHS device are installed with operating parameter sensors capable of remote data transmission, including Pt100 temperature sensors, pressure transmitters, and turbine flowmeters. Manual regulating valves are also configured to adjust the resistance of each branch. To ensure the reliability and reproducibility of the experimental results, the specific models and uncertainties (accuracies) of all sensors and data acquisition equipment used in this study are detailed in Table 4. The layout of the installed system monitoring components is shown in Figure 19.

**Figure 20:**
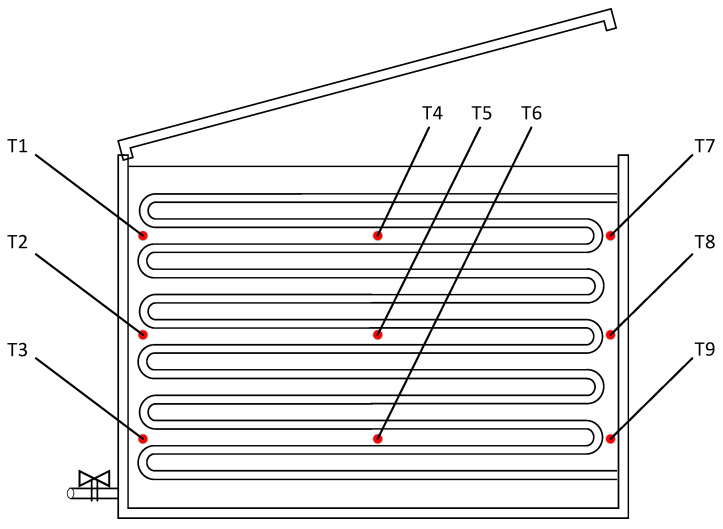
Arrangement of temperature measurement points within NP-LHS.

To accurately monitor the highly transient phase-change behavior and temperature distribution of the PCM, nine Pt100 temperature sensors were strategically placed inside the NP-LHS device. As illustrated in Figure 20, these sensors are arranged in a 3 × 3 spatial grid: they are distributed at three different height levels (top, middle, and bottom layers) and three horizontal positions (left side, center, and right side, between the tube bundles). The arithmetic mean of the readings from these nine sensors is calculated to represent the overall internal average temperature of the PCM during the charging and discharging cycles.

**Figure 21:**
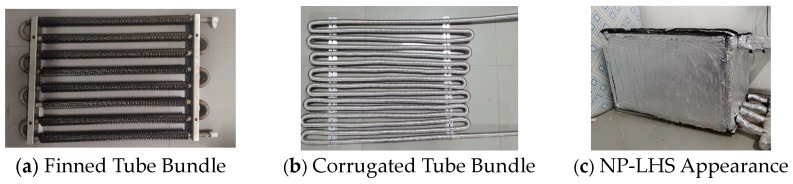
Main structure of phase-change energy storage device. Adapted with permission from Ref. [36]. Copyright 2026.

The main structure of the NP-LHS device is shown in Figure 21. The finned tube bundle is shown in Figure 21a, and the corrugated tube bundle is shown in Figure 21b. The device shell used in the experiment has parameters of 800 mm length, 400 mm width, and 500 mm height. The outer shell is wrapped with an insulation layer to reduce the natural heat dissipation of the equipment, as shown in Figure 21c.

### 4.2. Experimental Results and Discussion

#### 4.2.1. Comparative Analysis of Heat Storage Experimental Results

To evaluate the system-level dynamic performance, empirical charging tests for both the corrugated and finned tube LHS prototypes were executed under Control Logic 3. This specific mode entails the simultaneous activation of the thermal storage unit alongside the indoor fan coils. During these trials, the return water setpoint to the ASHP unit was strictly maintained at 45 °C. An analysis of the resulting thermal charging kinetics reveals that the total time required for complete liquefaction is highly comparable between the two geometries, with the finned tube module requiring 298 min, only marginally longer than the 280 min recorded for the corrugated configuration. However, it is noteworthy that the heat storage termination temperature of the finned tube storage device is 46 °C, which is 3 °C higher than that of the corrugated tube device. In the context of low-temperature ASHP heating, this 3 °C elevation in the terminal supply water temperature provides a higher sensible heat capacity for the fan coils, which is crucial for maintaining indoor thermal comfort during severe cold periods. The true superiority of the finned tube geometry emerges when evaluating the transient thermal response required to reach a functional average temperature of 43 °C. Under these conditions, the finned configuration requires merely 180 min—a charging cycle drastically faster than the corrugated alternative. Crucially, this accelerated heat transfer is achieved simultaneously with a substantially higher PCM volume fraction. By combining a denser thermal mass with remarkably swift energy absorption, the finned tube design delivers a decisively superior overall charging performance compared to the corrugated module.

#### 4.2.2. Comparative Analysis of Heat Release Experimental Results

We compared the heat release of the finned and corrugated tubes after charging them at 45 °C. The finned tube reached a peak latent heat extraction of 61.4% at a 20 °C indoor setting. The corrugated tube had a higher peak of 71.0% at 22 °C with a medium fan speed. The finned tube’s lower percentage is mainly because it holds more PCM. With a larger total mass, some of the material is farther away from the tubes, making it harder to extract all the heat within the test timeframe.

However, the finned tube was much better at keeping the room warm for a long time. It released heat for 179 min at an 18 °C setting, while the corrugated tube only lasted 109 min. Even with a slightly lower extraction rate, the finned design’s large energy reserve provides much longer indoor comfort during peak power hours.

#### 4.2.3. Comprehensive Performance Assessment and Power Regulation Capability

To provide a clearer and more intuitive understanding of the performance differences among the three tube configurations, a comprehensive comparison is summarized in Table 5. The table consolidates the key metrics from both the CFD numerical simulations (PCM volume fraction, charging/discharging durations) and the full-cycle experimental tests (maximum latent heat extraction rate, peak-shaving power reduction rate). It clearly demonstrates that the proposed finned tube NP-LHS device achieves the highest PCM filling rate and the best overall power demand regulation capability.

To elucidate the fundamental reasons behind the significant performance disparities among the three tube geometries, a detailed heat transfer analysis is required. The overall thermal resistance during the phase-change process in the NP-LHS device can be broadly divided into three components: the internal convective resistance of the HTF (R_int_), the conductive resistance of the tube wall (R_wall_), and the transient thermal resistance of the PCM side (R_pcm_), which involves both conduction and natural convection.

In the bare tube configuration, as the melting process initiates, a layer of liquid PCM forms around the tube. Because the organic PCM possesses an inherently low thermal conductivity, this growing liquid layer acts as a severe thermal insulator. Although buoyancy-driven natural convection develops within the liquid melt pool, it is insufficient to overcome the dominant conductive resistance (R_pcm_), resulting in an unacceptably long total melting time (691 min).

The corrugated tube attempts to enhance heat transfer by inducing fluid disturbance and macroscopic turbulence within the internal HTF flow, thereby effectively reducing the internal convective resistance (R_int_). However, because the primary thermal bottleneck in this system lies on the PCM side (R_pcm_ ≫ R_int_), improving the water-side heat transfer coefficient yields only marginal overall benefits. Consequently, the corrugated tube only reduces the melting time to 579 min, which remains impractical for rapid peak-shaving applications.

In stark contrast, the finned tube geometry addresses the root cause of the thermal bottleneck. The extended longitudinal metallic fins physically penetrate the insulating liquid PCM layer, effectively extending the highly conductive pathways deep into the solid PCM domain. This structural intervention drastically reduces the effective thermal resistance on the PCM side (R_pcm_) by shifting the dominant heat transfer mechanism from sluggish natural convection to rapid, extended-surface conduction. As a result, the finned tube accelerates the phase-change process dramatically, achieving complete melting in just 180 min, even while accommodating a significantly higher PCM volume fraction (92.5%).

### 4.3. Engineering Implications and Limitations of the Non-Pressurized Design

The NP-LHS device offers strong peak-shaving performance, but its non-pressurized design does come with some drawbacks compared to sealed systems.

The main thermal issue is the openable top cover. Even with 50 mm of insulation, this creates a thermal bridge that causes more heat loss than a fully sealed tank. Also, the PCM shrinks during solidification. Without pressure pushing the material against the tubes, tiny air gaps can form, increasing contact resistance. But as our tests show, the massive surface area of the finned tubes easily overcomes this problem and keeps charging and discharging fast.

Over many cycles, organic PCMs in unsealed tanks might suffer from oxidation or moisture absorption. Surprisingly, this is a major benefit of the open design. If the PCM degrades in a sealed tank, the whole unit must be replaced. With the NP-LHS, a bottom drain and an open top allow users to simply empty the old PCM and refill the tank cheaply. This also means the material can be swapped out for different seasons (like using a different melting point for summer cooling).

### 4.4. Validation of the Numerical Model

To ensure the reliability and accuracy of the present CFD simulations (employing the enthalpy-porosity method, the Boussinesq approximation, and the SIMPLEC algorithm), a quantitative validation was performed by comparing the numerical predictions with the experimental measurements obtained from the actual NP-LHS prototype coupled with the ASHP system.

Due to the complex internal structure of the LHS device and the highly transient nature of the phase-change process, directly measuring the local liquid fraction inside the tank is experimentally challenging. Therefore, the macroscopic heat storage duration (i.e., the time required for the PCM to fully melt) and the transient temperature evolution of the PCM were selected as the key validation metrics.

For the validation case, the experimental operating condition with a constant HTF inlet temperature of 45 °C and an inlet velocity of 0.1 m/s was selected. The numerical model of the finned tube configuration was simulated under identical boundary and initial conditions (initial PCM temperature of 39.5 °C).

The simulated transient temperature profile of the PCM exhibits a consistent trend with the experimental data throughout the entire heat storage process. In the initial sensible heating stage, the PCM temperature rises rapidly. During the latent heating stage, the temperature increase slows down significantly due to the absorption of latent heat. Finally, the temperature rises again as the sensible heating of the liquid PCM commences.

The experimental results indicated that the complete melting of the PCM (heat storage duration) required approximately 280 min. The CFD simulation predicted a complete liquefaction time (liquid fraction *β* = 1) of 293 min. The relative error between the simulated and experimental heat storage durations is calculated as follows:

$$Error=\frac{\left|t_{sim}-t_{exp}\right|}{t_{exp}}\times 100\%=\frac{\left|293-280\right|}{280}\times 100\%\approx 4.6\%$$

The maximum relative error between the numerical predictions and the experimental measurements is well within the acceptable engineering margin of 10%. The small discrepancy in melting times between the model and the experiment comes from three main sources:

(1) The simulation was perfectly adiabatic, whereas the actual tank experienced minor heat loss through its walls.

(2) Numerical inputs for PCM properties were constant, but in reality, these values fluctuate with temperature.

(3) Thermocouples measure data at discrete locations, which differs from the continuous volume average calculated in CFD.

Ultimately, the results align well enough to confirm that the model accurately captures the phase-change mechanics and can be used for system design.

## 5. Conclusions

Addressing the urgent need for efficient energy storage in the field of building heating, this paper designed and manufactured a novel NP-LHS. Its internal structure was optimized through numerical simulation, and an experimental platform coupled with an ASHP was built to systematically compare the heat storage and release characteristics of corrugated tube and finned tube over a full cycle. The main conclusions are as follows:

1. CFD numerical simulation based on the enthalpy-porosity method revealed the law of the influence of tube spacing on the advancement of the solid–liquid phase interface. It was found that when the tube spacing is controlled between 1.0 and 1.5 times the tube diameter, the liquefaction rate of the PCM and the heat storage duration achieve the best balance. After exceeding 1.5 times the tube diameter, the growth rate of the liquefaction rate slows down significantly, leading to a decrease in heat storage efficiency.

2. Both the simulation and experiments show that although the corrugated tube enhances convective heat transfer by inducing turbulence in the fluid inside the tube, in the non-pressurized shell-and-tube structure of this study, the finned tube exhibits better comprehensive performance due to its extended heat conduction area and higher PCM filling rate.

3. The volume proportion of PCM inside the finned tube device is as high as 92.5%, higher than the 88.2% of the corrugated tube device and the 77% of the bare tube device, significantly increasing the total energy storage capacity of the system

4. The experimental data show that the temperature rise rate of the finned tube device is significantly faster than that of the corrugated tube. To reach the same heat storage temperature (43 °C), the finned tube device takes only 180 min, shortening the time by about 35.7% compared to the corrugated tube device (280 min); the final heat storage temperature reached by the finned tube is higher (46 °C), indicating that it can absorb more heat within a limited off-peak electricity period.

5. Under the joint operation mode of “Heat Pump Heat Storage + Fan Coil Heat Release”, the finned tube storage device not only stores heat faster but also maintains the indoor target temperature for a longer time during the heat release stage. This indicates that in non-pressurized energy storage systems using low thermal conductivity materials like paraffin/fatty acids as media, using finned tubes to extend the surface area has more practical engineering value than using corrugated tubes to enhance turbulence inside the tube.

## Figures and Tables

**Table 1:** Thermophysical properties of the selected composite PCM.

Parameter	Symbol	Value	Unit
Melting temperature (peak)	*T_m_*	40.69	°C
Latent heat of fusion	*h_PCM_*	190	kJ/kg
Density (Solid)	*ρ_p_*_,*s*_	850	kg/m^3^
Density (Liquid)	*ρ_p_*_,*l*_	790	kg/m^3^
Thermal conductivity (Solid)	*λ_s_*	0.25	W/(m·K)
Thermal conductivity (Liquid)	*λ_l_*	0.20	W/(m·K)
Specific heat capacity	*C_p_*_,*p*_	1900	J/(kg·K)
Dynamic viscosity	*μ_p_*	0.003	N·s/m^2^

**Table 2:** Mode switching control of LHS.

No.	Mode	Valves Opened	Valves Closed	System Components Involved	Applicable Scenario
1	Conventional Operation Mode	1, 2, 3, 4, 7	5, 6	ASHP unit, Fan Coil Units	Heating demand exists, grid power is sufficient, and renewable energy generation is insufficient
2	Storage Device Independent Heat Storage Mode	1, 6, 7	2, 3, 4, 5	ASHP unit, Heat Storage Device	No heating demand, and renewable energy generation is sufficient during grid off-peak hours
3	Fan Coil and Storage Device Joint Operation Mode	1, 2, 3, 4, 6, 7	5	ASHP unit, Fan Coil Units, Heat Storage Device	Low heating demand, and renewable energy generation or power supply is sufficient
4	Storage Device Energy Release Mode	2, 3, 4, 5	1, 6, 7	Heat Storage Device, Fan Coil Units, Release Pump	Heating demand exists during grid peak hours, and the storage tank is sufficiently charged

**Table 3:** Parameters of experimental platform equipment.

Name	Model Quantities	Rated Parameters
Air-cooled heat pump units	1	cooling capacity: 18.5 kW heating capacity: 20.1 kW
FCU-C	2	cooling capacity: 7.2 kW heat capacity: 11.16 kW
FCU-K	1	cooling capacity: 4.63 kW heat capacity: 6.95 kW

**Table 4:** Specifications and measurement accuracies of the experimental equipment and sensors.

No.	Equipment/Sensor Name	Qty.	Model	Specifications and Accuracy
1	Temperature and humidity data logger	2	RC-4HC	Measurement accuracy: ±0.5 °C
2	Pt100 Temperature sensor	8	Pt100	Measurement accuracy: ±0.1 °C
3	Turbine flow transmitter	5	HR-LWGB-15	Accuracy class: ±0.1%
4	Pressure transmitter	7	SUP-P300	Accuracy class: 0.5
5	Solenoid valve	7	2W-200-20	Operating temperature range: −5~80 °C
6	Temperature data acquisition module	1	KHTH-TR-16	Communication standard: Isolated RS485, Standard MODBUS
7	Pressure and flow data acquisition module	1	KHAQ-16AI	Communication standard: Isolated RS485, Standard MODBUS
8	Programmable Logic Controller (PLC)	1	ST-20	Power consumption: 14 W Max available current: 300 mA
9	Paperless recorder	1	CTR-380-16	Accuracy class: 0.5

**Table 5:** Comprehensive performance comparison of LHS devices with different tube geometries.

Tube Geometry	PCM Volume Fraction (%)	Simulated Heat Storage Time (min)	Simulated Heat Release Time (min)	Experimental Max. Latent Heat Extraction Rate (%)	Experimental Max. Power Reduction Rate (%)
Bare Tube	77.0%	691	~238	N/A *	N/A *
Corrugated Tube	88.2%	579	293	71.0%	95.2%
Finned Tube	92.5%	576	500	61.4%	98.0%

Note: N/A * indicates that experimental system-level tests were not conducted for the bare tube configuration, as its preliminary evaluation was performed primarily through numerical simulations.

## Data Availability

The original contributions presented in this study are included in the article. Further inquiries can be directed to the corresponding authors.

## Nomenclature

NP-LHS	Non-pressurized shell-and-tube latent heat storage
LHS	Latent heat storage
PCM	Phase change material
HTF	Heat transfer fluid
ASHP	Air-source heat pump system
SHS	Sensible heat storage
TCHS	Thermochemical heat storage
TES	Thermal energy storage
DSC	Differential scanning calorimetry
*A*_1_	Inner wall surface area
*c_l_*	Specific heat capacity of liquid PCM
*c_s_*	Specific heat capacity of solid PCM
*h*	Phase change latent heat of PCM
*h*_1_	Inner wall convective heat transfer coefficient
*h*_2_	Outer wall convective heat transfer coefficient
*k*_1_	Overall heat transfer coefficient based on inner wall surface
*m_l_*_,*t*_	Mass of liquid PCM inside the device at time t
*m_s_*_,*t*_	Mass of solid PCM inside the device at time t
*Q*_1_	Total heat exchange amount between HTF and PCM
*Q*_1,*i*_	Heat exchange amount in the *i*-th differential segment
*Q*_2_	Heat exchange amount between PCM of different phase states
*Q*_3_	Heat exchanged inside liquid PCM
*Q*_4_	Heat exchanged inside solid PCM
*Q*_5_	Latent heat absorbed/released by PCM during phase change
*r_i_*	Inner radius of the tube
*r_o_*	Outer radius of the tube
*t*	Time
*T*_1,*i*_	Temperature of HTF in the *i*-th differential segment
*T* *_p_*_,*i*_	Temperature of PCM in contact with the outer wall in the *i*-th segment
*T_l_*_,*t*_	Temperature of liquid PCM at time t
*T_s_*_,*t*_	Temperature of solid PCM at time t
*δ*	Tube wall thickness
*λ*	Thermal conductivity of the tube wall
*τ*	Heat exchange duration
